# Celiac artery mesenteric fat measurement with endosonography (CAMEUS) reliably correlates with obesity and related comorbidities

**DOI:** 10.1093/gastro/goae039

**Published:** 2024-04-26

**Authors:** Fateh Bazerbachi, Serge Baroud, Michael J Levy, Daniel B Maselli, Eric J Vargas, Aliana Bofill-Garcia, Ryan J Law, Vinay Chandrasekhara, Andrew C Storm, Ferga C Gleeson, Elizabeth Rajan, Prasad G Iyer, Kymberly D Watt, Barham K Abu Dayyeh

**Affiliations:** CentraCare, Interventional Endoscopy Program, St Cloud Hospital, St. Cloud, MN, USA; Division of Gastroenterology, Hepatology and Nutrition, University of Minnesota, Minneapolis, MN, USA; Department of Internal Medicine, MetroHealth Medical Center and Case Western Reserve University, Cleveland, OH, USA; Division of Gastroenterology and Hepatology, Mayo Clinic, Rochester, MN, USA; Division of Gastroenterology and Hepatology, Mayo Clinic, Rochester, MN, USA; Division of Gastroenterology and Hepatology, Mayo Clinic, Rochester, MN, USA; Division of Gastroenterology and Hepatology, Mayo Clinic, Rochester, MN, USA; Division of Gastroenterology and Hepatology, Mayo Clinic, Rochester, MN, USA; Division of Gastroenterology and Hepatology, Mayo Clinic, Rochester, MN, USA; Division of Gastroenterology and Hepatology, Mayo Clinic, Rochester, MN, USA; Division of Gastroenterology and Hepatology, Mayo Clinic, Rochester, MN, USA; Division of Gastroenterology and Hepatology, Mayo Clinic, Rochester, MN, USA; Division of Gastroenterology and Hepatology, Mayo Clinic, Rochester, MN, USA; Division of Gastroenterology and Hepatology, Mayo Clinic, Rochester, MN, USA; Division of Gastroenterology and Hepatology, Mayo Clinic, Rochester, MN, USA

**Keywords:** endoscopic ultrasonography, upper endoscopy, nonalcoholic fatty liver disease, obesity, metabolic syndrome

## Abstract

**Background:**

Visceral fat represents a metabolically active entity linked to adverse metabolic sequelae of obesity. We aimed to determine if celiac artery mesenteric fat thickness can be reliably measured during endoscopic ultrasound (EUS), and if these measurements correlate with metabolic disease burden.

**Methods:**

This was a retrospective analysis of patients who underwent celiac artery mesenteric fat measurement with endosonography (CAMEUS) measurement at a tertiary referral center, and a validation prospective trial of patients with obesity and nonalcoholic steatohepatitis who received paired EUS exams with CAMEUS measurement before and after six months of treatment with an intragastric balloon.

**Results:**

CAMEUS was measured in 154 patients [56.5% females, mean age 56.5 ± 18.0 years, body mass index (BMI) 29.8 ± 8.0 kg/m^2^] and was estimated at 14.7 ± 6.5 mm. CAMEUS better correlated with the presence of non-alcoholic fatty liver disease (NAFLD) (*R*^2^ = 0.248, *P *<* *0.001) than BMI (*R*^2^ = 0.153, *P *<* *0.001), and significantly correlated with metabolic parameters and diseases. After six months of intragastric balloon placement, the prospective cohort experienced 11.7% total body weight loss, 1.3 points improvement in hemoglobin A1c (*P *=* *0.001), and a 29.4% average decrease in CAMEUS (−6.4* *±* *5.2 mm, *P *<* *0.001). CAMEUS correlated with improvements in weight (*R*^2^ = 0.368), aspartate aminotransferase to platelet ratio index (*R*^2^ = 0.138), and NAFLD activity score (*R*^2^ = 0.156) (all *P *<* *0.05).

**Conclusions:**

CAMEUS is a novel measure that is significantly correlated with critical metabolic indices and can be easily captured during routine EUS to risk-stratify susceptible patients. This station could allow for EUS access to sampling and therapeutics of this metabolic region.

## Introduction

According to data from the National Health and Nutrition Examination Survey, the age-adjusted prevalence of obesity has increased from 30.5% to 42.4% over the past two decades [1]. Obesity increases the risk of metabolic disease, many malignancies, and various infections, including coronavirus disease 2019 [2]. Visceral obesity, in particular, has been linked to the development of an insulin-resistant and pro-inflammatory state, a harbinger of metabolic syndrome and its detrimental effects on health [3, 4].

Key metabolic risk factors implicated in the development of obesity and metabolic syndrome also increase the risk of non-alcoholic fatty liver disease (NAFLD), a condition of excessive adiposity in the liver. Patients with obesity and diabetes are particularly susceptible to NAFLD, with reported prevalence exceeding 95% in severely obese patients [5, 6]. Roughly one-quarter of patients with NAFLD will develop non-alcoholic steatohepatitis (NASH), the inflammatory subtype that promotes the progression to cirrhosis, portal hypertension, and hepatocellular carcinoma. Because of this accelerated cadence towards end-stage liver disease and its complications, there is considerable interest in identifying patients at higher risk for NASH and early fibrosis, to stratify and identify those warranting closer surveillance and more aggressive therapy. Presently, the gold standard diagnostic modality for NASH relies on histology and thus liver biopsy, which is hampered by invasiveness, patient inconvenience, and sampling errors, among other factors [7, 8].

Given the increasing implementation of endoscopic modalities in the diagnosis, risk stratification, and therapy of a variety of gastrointestinal and hepatobiliary diseases, endoscopy may fill management gaps in patients with NAFLD [9–13]. For example, echoendoscopes can intimately evaluate regions of interest relevant to NAFLD and metabolic conditions, such as visceral fat deposits, which may be more accurate compared with external ultrasound-based techniques. Furthermore, endoscopic ultrasound (EUS)-examined stations are reproducible, regardless of gastric motility or intestinal distention, and imaging artifacts can be minimized by suctioning air and/or water immersion during the procedure. This may not be the case for external imaging modalities, such as computed tomography (CT) or magnetic resonance imaging (MRI). Therefore, EUS is potentially well-positioned to investigate visceral adiposity by measuring the fat deposits and perhaps readily sampling this innermost plane [14].

This study aimed to determine if aspects of visceral adiposity can be reliably observed and measured during routine EUS assessment of the gastrohepatic ligament, through estimation of the celiac artery mesenteric fat thickness, referred to here as the celiac artery mesenteric fat measurement with endosonography (CAMEUS) [14]. Additionally, the study aimed to determine if CAMEUS correlates with metabolic disease indices and hallmarks of NAFLD.

## Materials and methods

This study included a retrospective arm with a validation prospective trial.

### Retrospective arm

This was a single-center retrospective study IRB (21–004112) from Mayo Clinic Rochester (MN, USA). Individuals who underwent EUS from February 2017 to May 2020 with CAMEUS measurement by a single experienced endosonographer (B.K.A.D.) were included in this study. The exclusion criteria included patients with a cancer diagnosis, chronic pancreatitis, and hepatic steatosis other than NAFLD. Data were retrospectively collected for the following characteristics: age, sex, ethnicity/race, body mass index (BMI), diabetes mellitus (DM), hypertension, hyperlipidemia, hypertriglyceridemia, and obstructive sleep apnea. Laboratory values within one year of CAMEUS measurement of cholesterol and triglycerides levels, low-density lipoprotein (LDL), high-density lipoprotein (HDL), hemoglobin A1C (HbA1C), platelet count, alanine aminotransferase, aspartate aminotransferase, and alkaline phosphatase were also retrieved. Weight trends, as well as serial laboratory tests and CAMEUS measurements, were also captured, and those with less than 5% total body weight loss (TBWL) fluctuation were included. Metabolic syndrome was determined according to the definition from the International Diabetes Federation: central obesity was defined according to the documented waist circumference or assumed if BMI was over 30 kg/m^2^ [15]. If the patient had central obesity, then the presence of at least two of the four factors was investigated to establish the diagnosis of metabolic syndrome (raised triglycerides or specific treatment for this lipid abnormality, reduced HDL cholesterol or specific treatment for this lipid abnormality, raised blood pressure or treatment of previously diagnosed hypertension, and raised fasting plasma glucose or previously diagnosed type 2 DM) [15].

For patients whose waist circumference measurement was not available, and BMI was less than 30 kg/m^2^, central obesity could not be excluded, and patients were labeled as not having metabolic syndrome only if three of the aforementioned four factors were not met. In all other cases where there were insufficient data to either diagnose or exclude metabolic syndrome, the patient’s metabolic syndrome status was labeled as unknown. After excluding patients with hepatic steatosis other than NAFLD, patients were considered to have NAFLD if it was documented in a gastroenterology or endocrinology clinical note and/or qualitative radiologic evidence of hepatic steatosis by abdominal ultrasound, EUS, CT, MRI, magnetic resonance elastography (MRE), and/or liver biopsy within one year of CAMEUS measurement provided that other potential causes of secondary hepatic steatosis were not present [8].

### CAMEUS

Celiac artery mesenteric fat measurement with endosonography (CAMEUS) was employed to quantify visceral adiposity in the celiac artery region. Patients were under monitored anesthesia care or general endotracheal anesthesia and placed in the left lateral decubitus position. EUS examinations were performed with a curvilinear array echoendoscope (GF-UCT180; Olympus America, Center Valley, PA, USA).

### Preparation

The stomach was thoroughly desufflated to optimize image quality and reduce potential measurement distortion. A water-filled balloon attached to the tip of the EUS scope was used to further improve acoustic coupling and visualization.

***Celiac trunk identification*:** The EUS probe was carefully positioned to identify the origin of the celiac trunk from the abdominal aorta. Key anatomical landmarks may be used to facilitate identification, such as the superior mesenteric artery and the left renal vein.

***Image acquisition*:** The probe was torqued to obtain a clear sagittal/linear view of the celiac trunk without angulation, ensuring optimal visualization of its branching point. The image was frozen on the EUS screen to facilitate precise measurement.

### CAMEUS measurement

**Figure 1.**
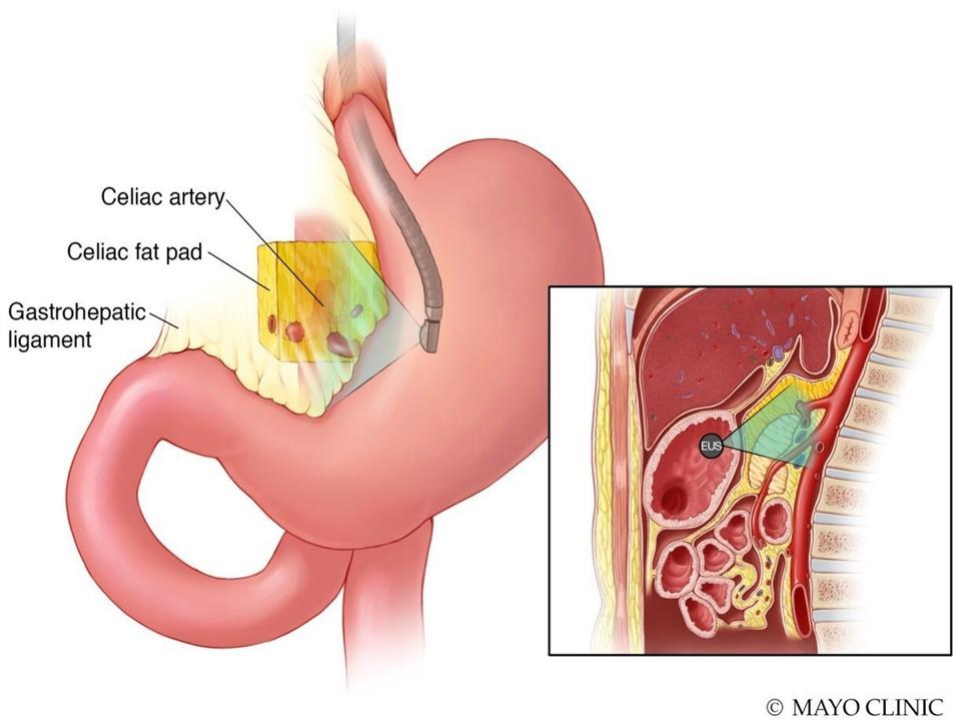
Endoscopic ultrasound (EUS) approach to assess celiac fat pad thickness

**Figure 2.**
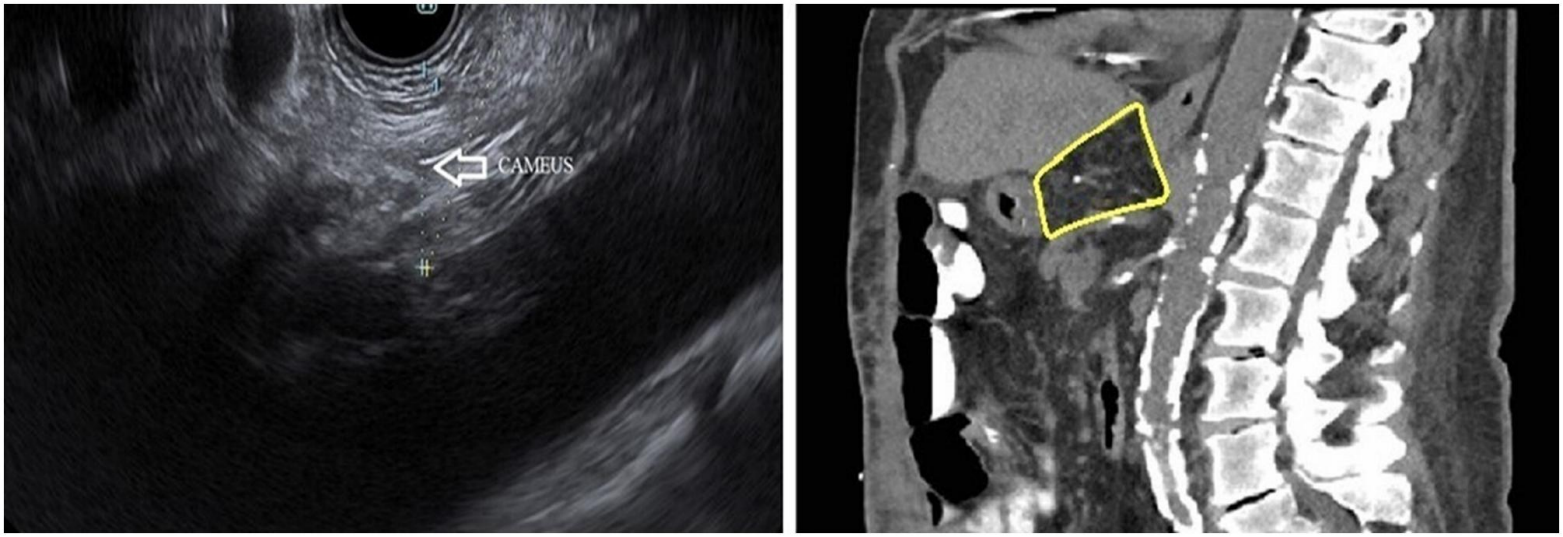
Endoscopic ultrasound estimation of celiac artery mesenteric fat thickness (left) and computed tomography scan correlate of celiac artery mesenteric fat thickness (right). CAMEUS = celiac artery mesenteric fat measurement with endosonography.

Using the EUS system's measurement tools, a perpendicular line was drawn from the celiac trunk's branching point to intersect the hyperechoic muscularis propria layer of the gastric wall, precisely at the point where the EUS transducer made contact. The length of this perpendicular line was measured and recorded in millimeters as the CAMEUS measurement (Figures 1 and 2). Data points were analysed for correlations between the metabolic indices, NAFLD, and CAMEUS.

### Prospective validation arm

The prospective validation arm was a single-center, prospective study conducted at Mayo Clinic Rochester (MN, USA) from October 2016 to March 2018. The study examined the effects of intragastric balloon therapy on improvements in NASH and metabolic parameters and results previously detailed [13]. CAMEUS measurements were performed as a sub-analysis in this cohort and results were not previously analysed or included in the aforementioned study. Patients with evidence of hepatic steatosis and failed lifestyle interventions were included and patients with other causes of liver steatosis were excluded [13]. Twenty-one included patients underwent paired EUS-guided fine needle biopsies of the liver, placement of single fluid-filled Orbera IGB (Apollo Endosurgery, Austin, TX, USA), MRE for liver stiffness (MRE-LS), MRI proton density fat fraction (MRI-PDFF), and CAMEUS measurements prior to and following 6 months of treatment with IGB for weight loss [13]. This provided 42 data points of CAMEUS measurements and corresponding metabolic indices implicated in NAFLD, liver biochemistries, NAFLD activity score (NAS), MRE-LS, and MRI-PDFF measurements, which were analysed by linear regression for correlations [13]. Corrections were made for repeated measurements.

### Statistical analysis

Data for both study arms are expressed as the mean* *±* *standard deviation (SD), median [interquartile range (IQR)], or number (frequency). Comparative statistical analysis was performed using the paired *t*-test for continuous variables as well as linear and logistic regression analysis for correlations using JMP Pro version 14.1.0 (SAS Institute, Cary, NC, USA). A *P*-value of <0.05 was considered statistically significant.

## Results

### Retrospective cohort

The baseline demographics, comorbidities, and relevant laboratory values of the retrospective cohort are shown in Table 1. The retrospective cohort included 154 patients: 56% were females with a mean age of 56.5 ± 18.0 years and a mean CAMEUS measurement of 14.7 ± 6.5 mm. Weight subgroup distribution in this cohort was as follows: 3% underweight (BMI <18.5 kg/m^2^), 27% normal weight (BMI 18.5–24.9 kg/m^2^), 27% overweight (BMI 25–29.9 kg/m^2^), 20% class I obesity (BMI 30–34.9 kg/m^2^), 9% class II obesity (BMI 35–39.9 kg/m^2^), and 14% class III obesity (BMI ≥40 kg/m^2^) [16]. While metabolic syndrome was present in 27.9% and absent in 63.0%, it could not be determined in 9.1% of patients. NAFLD was present in 75 patients (48.7%), with radiologic evidence of steatosis by ultrasound (14.7%), CT (34.7%), MRI (20.0%), MRE (4.0%), and/or on histology (26.7%).

**Table 1.** Baseline characteristics and clinical data in the retrospective cohort (*n *=* *154)

Variable	Values
Age, years, mean* *±* *SD	56.5 ± 18.0
Female, *n* (%)	87 (56.5)
Weight, kg, mean* *±* *SD	85.7 ± 26.3
BMI, kg/m^2^, mean* *±* *SD	29.8 ± 8.0
Prediabetes, *n* (%)	25 (16.2)
Type 2 diabetes mellitus, *n* (%)	42 (27.3)
Type 1 diabetes mellitus, *n* (%)	2 (1.3)
Hypertension, *n* (%)	83 (53.9)
Hyperlipidemia, *n* (%)	77 (50.0)
Hypertriglyceridemia, *n* (%)	42 (27.3)
Obstructive sleep apnea, *n* (%)	40 (26.0)
Metabolic syndrome, *n* (%)	
Present	43 (27.9)
Absent	97 (63.0)
Undetermined	14 (9.1)
Hemoglobin A1c^a^, %, mean* *±* *SD	6.1 ± 1.2
Total cholesterol^b^, mg/dL, mean* *±* *SD	170.6 ± 47.0
LDL^c^, mg/dL, mean* *±* *SD	93.5 ± 39
HDL^b^, mg/dL, mean* *±* *SD	52.6 ± 22.8
Triglycerides^b^, mg/dL, mean* *±* *SD	136.6 ± 82.5
ALT for male^d^, U/L, mean* *±* *SD	63.9 ± 102.6
ALT for female^e^, U/L, mean* *±* *SD	42.7 ± 47.3
AST for male^f^, U/L, mean* *±* *SD	51.2 ± 78.6
AST for female^e^, U/L, mean* *±* *SD	44.8 ± 69.1
ALP for male^g^, U/L, mean* *±* *SD	133.5 ± 148.9
ALP for female^h^, U/L, mean* *±* *SD	117.2 ± 69.7
APRI^i^, mean* *±* *SD	0.5 ± 0.9
NAFLD, *n* (%)	75 (48.7)
CAMEUS, mm, mean* *±* *SD	14.7 ± 6.5

a Due to the missing data, the total is 95.

b Due to the missing data, the total is 114.

c Due to the missing data, the total is 113.

d Due to the missing data, the total is 61.

e Due to the missing data, the total is 78.

f Due to the missing data, the total is 64.

g Due to the missing data, the total is 65.

h Due to the missing data, the total is 79.

i Due to the missing data, the total is 142.

ALT = alanine aminotransferase; ALP = alkaline phosphatase; APRI = aspartate aminotransferase-to-platelet ratio index; AST = aspartate aminotransferase; BMI = body mass index; CAMEUS = celiac artery mesenteric fat measurement with endosonography; HDL = high-density lipoprotein; LDL = low-density lipoprotein; NAFLD = non-alcoholic fatty liver disease; SD = standard deviation.

**Figure 3.**
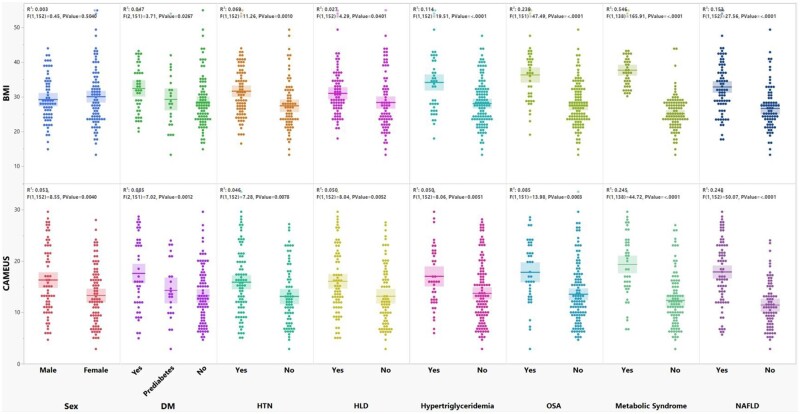
CAMEUS and BMI association with NAFLD and various metabolic indices and diseases are presented with box plot diagrams. BMI = body mass index; CAMEUS = celiac artery mesenteric fat measurement with endosonography; DM = diabetes mellitus; HLD = hyperlipidemia; HTN = hypertension; NAFLD = nonalcoholic fatty liver disease; OSA = obstructive sleep apnea.

**Figure 4.**
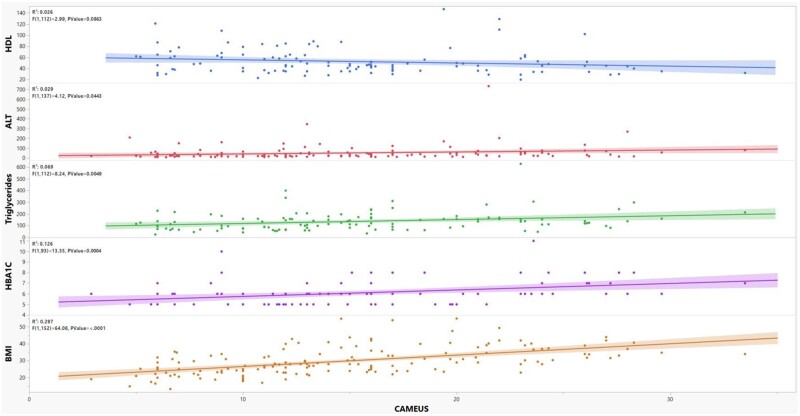
Scatter plots of CAMEUS association with BMI, various metabolic indices, and liver biochemistries. ALT = alanine aminotransferase; BMI = body mass index; HbA1c = glycated hemoglobin; HDL = high-density lipoprotein.

CAMEUS had a stronger correlation with NAFLD (*R*^2^ = 0.248, *P *<0.001) than BMI (*R*^2^ = 0.153, *P *<0.001) (Figure 3). A simple linear regression model showed that CAMEUS significantly correlated with metabolic syndrome (*R*^2^ = 0.245), as well as several obesity-related comorbidities, including type 2 DM and prediabetes (*R*^2^ = 0.085), obstructive sleep apnea (*R*^2^ = 0.085), hypertriglyceridemia (*R*^2^ = 0.050), hyperlipidemia (*R*^2^ = 0.050), and hypertension (*R*^2^ = 0.046), all *P *<* *0.05 (Figure 3). Additionally, CAMEUS significantly correlated with objective measurements relevant to obesity, NAFLD, and other metabolic comorbidities, including weight (*R*^2^ = 0.374), BMI (*R*^2^ = 0.297), HbA1C (*R*^2^ = 0.126), triglycerides (*R*^2^ = 0.069), alanine aminotransferase (*R*^2^ = 0.029), all *P *<0.05 (Figure 4). Although not statistically significant, there was a correlation trend between CAMEUS and HDL (inverse relationship), aspartate aminotransferase to platelet ratio index, aspartate aminotransferase, and LDL. In subgroup analysis, CAMEUS had a stronger correlation with NAFLD in males (*R*^2^ = 0.310, *P *<0.001) compared with females (*R*^2^ = 0.202, *P *<0.001).

A multivariate analysis using a logistic regression model including CAMEUS, age, sex, BMI, weight, DM, hypertension, hyperlipidemia, hypertriglyceridemia, and metabolic syndrome was performed to evaluate predictive factors for NAFLD. The model showed that CAMEUS is a predictive factor for NAFLD (OR: 1.15, 95% CI: 1.055–1.270).

### Prospective validation arm

**Figure 5.**
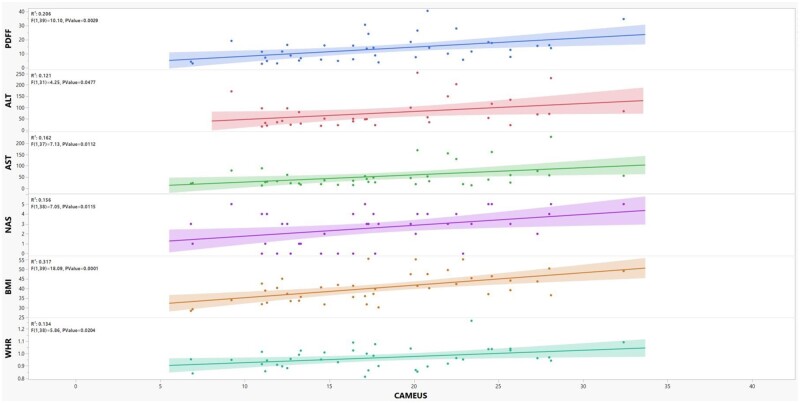
Scatter plots of CAMEUS association with measures of obesity, liver biochemistries, non-alcoholic fatty liver disease activity score (NAS), and magnetic resonance imaging of proton density fat fraction (MRI-PDFF). ALT = alanine aminotransferase; AST = aspartate aminotransferase; BMI = body mass index; CAMEUS = celiac artery mesenteric fat measurement with endosonography; WHR = waist-to-hip ratio.

A total of 21 patients participated in the prospective study. At the time of intragastric balloon removal, patients lost an average of 11.7% of their total body weight (range: −0.1%, 32.5%) at 6 months. This resulted in a 29.4% (range: −27.05%, 59.65%) average decrease in mesenteric fat thickness (−6.4* *±* *5.2 mm, *P *<0.0001). With this change, the HbA1c improved by 1.3 (7.5 ± 1.6 vs 6.3 ± 1.2, *P *=* *0.001) and NAS improved from a median of 4 to 1 with 73% of patients achieving 2 or more points improvement in NAS (*P *<0.001). CAMEUS significantly correlated with weight loss (*R*^2^ = 0.368), BMI (*R*^2^ = 0.317), waist-to-hip ratio (*R*^2^ = 0.134), aspartate aminotransferase to platelet ratio index (*R*^2^ = 0.138), aspartate aminotransferase (*R*^2^ = 0.162), alanine aminotransferase (*R*^2^ = 0.121), NAS (*R*^2^ = 0.156), and MRI-PDFF (*R*^2^ = 0.206) (all *P *<* *0.05; Figure 5). MRE-LS was not significantly correlated with CAMEUS, although a correlation trend was observed (*R*^2^ = 0.052, *P *=* *0.217).

## Discussion

To the best of our knowledge, this is the first study to propose both a novel anthropometric measurement to estimate visceral fat deposits and a novel technique to estimate it based on the celiac artery mesenteric fat thickness measurement. Further, in this study, CAMEUS measurement correlated with key metabolic indices implicated in obesity, metabolic syndrome, and NAFLD. We also show that CAMEUS change was also associated with improvements in the metabolic parameters, radiologic findings, and histologic features of NAFLD following weight loss after intragastric balloon placement.

The metabolic syndrome includes a conglomerate of several metabolic indices such as dyslipidemia, hypertension, type 2 DM, and central obesity [15, 17]. Visceral adiposity, which is implicated in metabolic syndrome and NAFLD, is not specifically part of the diagnostic criteria of metabolic syndrome, and lacks reproducible and readily accessible estimation measures, and noninvasive sampling. We showed that aspects of visceral adiposity can be reliably estimated by CAMEUS during routine EUS, and postulated that it may be targeted in the future for sampling by EUS-guided techniques [18, 19].

In the retrospective arm, CAMEUS correlated with components of the metabolic syndrome including BMI, type 2 DM, hypertension, hypertriglyceridemia, HDL level (negative correlation), and metabolic syndrome as an entity. These statistically significant correlations are relevant to establishing CAMEUS as a novel anthropometric parameter, which can be incorporated into future studies when its acquisition is facilitated by a study design. Also, notably CAMEUS had a significantly stronger correlation than BMI with the diagnosis of NAFLD.

The prospective validation arm showed improvement in critical metabolic indices and evaluated the dynamic histologic changes in response to weight loss after intragastric balloon placement. We now provide CAMEUS measurements and changes in these patients and compare them with previously reported liver stiffness and hepatic fat content assessments [13]. CAMEUS significantly correlated with waist-to-hip ratio, BMI, liver chemistries, and aspartate aminotransferase to platelet ratio index. We have previously shown that percent total body weight loss achieved post-intervention did not correlate with improvement in NAS [13], and a similar observation has been described in the bariatric surgery literature [20]. CAMEUS change, on the other hand, significantly correlated with improvements in NAS. Therefore, we demonstrated that not only does CAMEUS strongly correlate with the presence of NAFLD, better than BMI, but also the change in CAMEUS will significantly correlate with a change in NASH activity. This correlation may be related to the fact that visceral adipose deposition, which poorly correlates with BMI [21–23], leads to defective and swollen adipocytes secreting greater amounts of inflammatory cytokines such as interleukin-6 and tumor necrosis factor-α and excessive fatty acids but less of the protective fat-derived hormones like adiponectin that play a crucial role in protecting against insulin resistance and obesity [24, 25]. Also, given the anatomical location of the visceral adipocytes and their venous drainage to the liver, hepatocytes are exposed to a higher bulk of fatty acids and pro-inflammatory molecules, thereby increasing fat production and deposition in the liver and stimulating further gluconeogenesis contributing to the development and progression of NAFLD [26, 27].

Early detection of worsening hepatic steatosis is clinically important and may lead to early intervention to halt the progression of steatohepatitis and fibrosis. The current gold standard for diagnosis and risk stratification depends on biopsied tissue which is both invasive and inconvenient [28]. The identification of other surveillance modalities for the follow-up of these patients noninvasively is therefore desired. Transient elastography and MRE-LS are useful noninvasive tools in predicting hepatic fibrosis and in the risk stratification of NAFLD patients even in the early stages of the disease [29–31]. In our study, MRE-LS was not significantly correlated with CAMEUS, although a correlation trend was observed (*R*^2^ = 0.052, *P *=* *0.217). Nonetheless, CAMEUS did correlate with MRI-PDFF (*R*^2^ = 0.206, *P *=* *0.003), another noninvasive modality utilized in assessing liver fat content that has been investigated for its potential application in assessing treatment response in NASH [32, 33]. EUS provides an advantage for patients who are already undergoing endoscopic procedures for a wide variety of pathologies and in patients with liver disease for screening purposes or surveillance for their varices. Another important advantage of EUS is the ability to not only assess the mesenteric fat pad for risk stratification but also allow an access window to sample the visceral fat for lipidomic profiling.

Our study has several shortcomings warranting acknowledgment. First, the retrospective arm displays inherent limitations, such as missing data points for some patients and the diagnosis of NAFLD based on different available modalities and not exclusively liver biopsy, the gold standard. However, only patients undergoing routine EUS with concomitant CAMEUS measurements performed by a single experienced endoscopist were included consecutively to minimize selection and measurement bias. Second, the prospective arm was an open-label single-arm study with no control group, the duration of follow-up did not allow the ascertainment of response durability, and mortality outcomes were not investigated [13]. Lastly, cross-sectional radiologic correlates to CAMEUS are lacking, as the region of interest could not be reliably reproduced by CT or MRI techniques (data not shown).

## Conclusions

CAMEUS is a novel anthropometric measure that significantly correlates with critical metabolic indices implicated in NAFLD and can be collected during routine endosonography to risk-stratify susceptible patients and monitor disease progression in those afflicted by NAFLD. Given the expanding field of bariatric endoscopy in obesity management [11, 34, 35], this new tool merits future studies to investigate its role in risk-stratifying patients with metabolic syndrome and in NAFLD natural history, pathogenesis, and management.

## Authors’ Contributions

B.K.A.D. contributed to the conception and design of the study, data analysis, and drafting of the article. F.B. contributed to the conception and design of the study, data abstraction, and drafting of the article. S.B. contributed to the design of the study, data abstraction, data analysis and interpretation, and drafting of the article. M.J.L. contributed to the drafting of the article. D.B.M. contributed to the drafting of the article. E.J.V., A. B-G., R.J.L., V.C., A.C.S., F.C.G., E.R., P.G. I., and K.D.W. contributed to the critical revision of the article. All authors have read and approved the final version of the manuscript.
